# Survey of food offered to United Kingdom haemodialysis patients attending for dialysis sessions in main dialysis centres and satellite units and international comparison

**DOI:** 10.1186/s41100-023-00466-3

**Published:** 2023-02-06

**Authors:** Andrew Davenport

**Affiliations:** UCL Department of Nephrology, Royal Free Hospital, University College London, Rowland Hill Street, London, NW3 2PF UK

**Keywords:** Haemodialysis, Nutrition, Food, Malnutrition, Elderly

## Abstract

**Background:**

Haemodialysis (HD) patients are at increased risk of frailty, sarcopenia and protein energy wasting, all associated with increased mortality. Most of the dialysis day is taken up with travelling to and from dialysis centres and dialysis treatment. The International Society of Nutrition and Metabolism (ISNM) recommend that meals or supplements should be part of standard clinical practice when patients attending for dialysis.

**Results:**

We surveyed adult UK centres to determine the provision of food to dialysis patients in the United Kingdom (UK). A hot meal was provided by six (8.7%) of the 69 UK adult units, although 16 (23.2%) main centres would potentially provide meals to a restricted number of malnourished patients. Forty-seven (68.1%) centres provided sandwiches, although this was restricted in eight main centres, and 26.2% of units did not provide sandwiches to patients in their satellite dialysis centres. Biscuits were the only nutrition routinely offered in 15 (21.7%) of the main dialysis units, 41.3% of satellite units. Meals were more likely to be offered in Northern Ireland and Scotland compared to England, and 38% of the main dialysis units in England, and 58% of their satellite centres did not routinely offer patients a sandwich compared to none or one centre in Wales, Scotland and Northern Ireland.

**Conclusions:**

Despite an increasing older, more frail dialysis population in the UK, food provision for dialysis patients has reduced, particularly in England, with < 10% of centres routinely offering hot food, and > 50% of dialysis units now only offering biscuits to their satellite dialysis patients.

## Introduction

The demographics of the haemodialysis (HD) population in Western Europe, North America and Japan has changed over the past 20 years, with increasing numbers of elderly patients with chronic kidney disease (CKD), and additional co-morbidities now receiving HD treatments [1]. These patients are at increased risk of frailty, sarcopenia and protein energy wasting, all of which are associated with increased mortality [2–5].

Muscle loss occurs not only by protein deficiency, but also by energy deficiency [6], and as such dietary intake is important in preventing sarcopenia and protein energy wasting. Dialysis patients are advised to follow a restrictive diet to reduce sodium, potassium and phosphate intake, and moreover may have additional protein losses in the urine and with dialysis [7, 8]. As such it has been recommended that dialysis patients eat 0.9 to 1.2 g/kg/day in Japan [6], and 1.0–1.2 k/g/day by the Kidney Disease Improving Global Outcomes Initiative (KDIGO), along with 25–35 kcal/kg/day [9]. The International Society of Renal Nutrition and Metabolism (ISRNM) recommended that meals or supplements should be considered as a part of the standard-of-care practice for patients attending for haemodialysis treatments, to help improve patients reaching nutritional targets [10].

Although the standard haemodialysis session is 4 h, many patients spend additional time travelling to and from the dialysis centre, waiting for the fistula needle sites to stop bleeding, and time to recover from dialysis [11], so may be away from their home for much longer. As dietary intake may well be suboptimum in many patients due to a variety of reasons, including poor appetite status, low diet quality, high diet monotony index, along with psychosocial and financial barriers [12], thus providing meals or supplements during haemodialysis may be an effective strategy to improve nutritional status [10]. As the pattern of practice in the UK has changed, with many more patients now dialysing in satellite dialysis centres, rather than in hospital dialysis units, we wished to determine current practice as to whether centres followed ISRNM recommendations that meals and supplements remain as part of the standard-of-care practice for patients attending for haemodialysis treatments.

## Methods

All adult dialysis centres in the United Kingdom (UK), including England, Wales, Scotland, and Northern Ireland were contacted and asked about foodstuffs offered to patients when they attended for outpatient haemodialysis treatments. To make an international comparison, nephrologists managing dialysis centres around the world were contacted and requested to provide information on local practices on the provision of food when patients attended for dialysis sessions.

### Statistics

Data are expressed as integer and percentage, with Chi-square analysis (X^2^) used for categorical data, and all analyses appropriately adjusted for small numbers and repeated analyses. Statistical analysis was performed using GraphPad Prism (version 9.4, Graph Pad, San Diego, CA, USA), Statistical Package for Social Science version 27.0 (IBM Corporation, Armonk, New York, USA). Statistical significance was taken at or below the 5% level.

### Ethics

This audit complied with UK National research Ethics (NRES) guidelines for clinical practice development and audit and did not require additional NRES approvals. All data were appropriately anonymised.

## Results

**Fig. 1:**
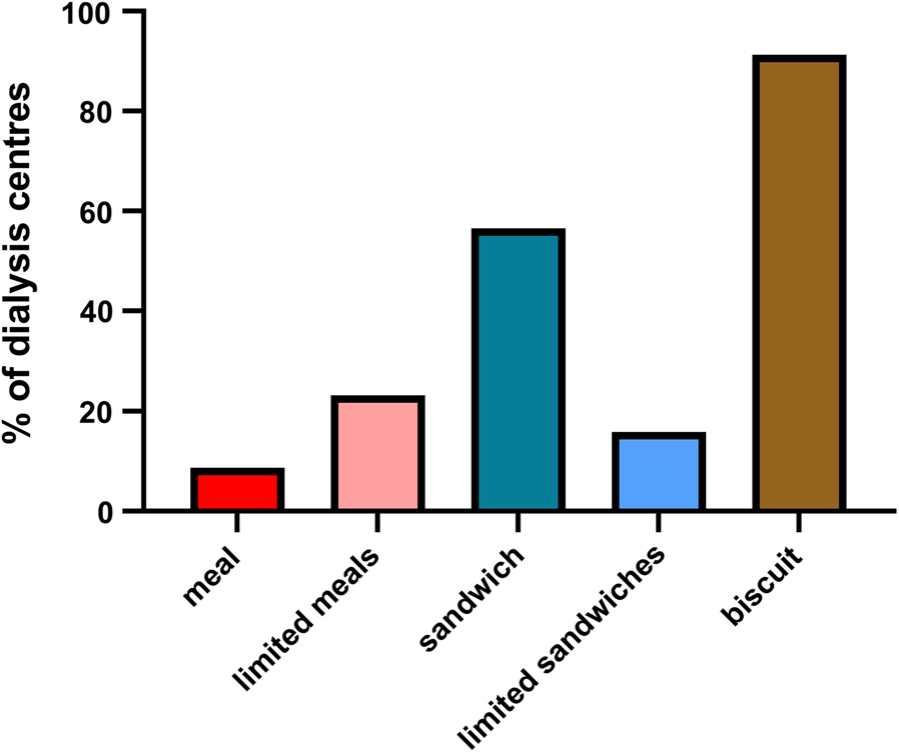
Routine provision of food in UK adult main haemodialysis centres. Restricted—not all patients attending for offered meal or sandwich

Replies were obtained from all 69 adult UK dialysis centres. Almost all centres provided patients with some food, although more centres provided sandwiches rather than meals (Fig. 1). Six centres (8.7%) regularly offered hot food, five offering a meal and one centre soup only. Hot food was provided at five main hospital dialysis centres (7.2%), and at two satellite units, one of which provided soup. Most of these centres only offered hot food to the morning and afternoon shifts in the main centre. Thus, patients dialysing in satellite units were less likely to be offered hot food (X^2^ 4.9, *p* = 0.03). Sixteen additional centres (23.2%) reported that although they did not routinely offer meals to patients, they would occasionally or rarely provide meals for patients with malnutrition or other nutritional issues in their main centre, as directed by advice for renal trained dietitians.

The majority of dialysis centres offered patients a sandwich (47 (68.1%)). However, this was restricted in three main centres to only patients who had been reviewed by dietitians and found to have poor nutritional intake. Three centres offered a snack box containing a sandwich, but this was again generally restricted to a minority of patients and typically directed by dietitian advice. Two of the main dialysis centres did not offer sandwiches to patients dialysing in the third or twilight shift. Three centres had temporarily withdrawn sandwiches during the Covid-19 pandemic. Eleven dialysis units (26.2%), which provided sandwiches to some patients in their main centre, did not provide sandwiches for those patients dialysing at one or more their satellite units. In addition to sandwiches, four main centres (5.8%) provided toast, predominantly to the morning shift, one centre provided popcorn or a slice of cake, and another cake when sandwiches were withdrawn due to Covid-19.

Nearly, all dialysis units routinely offered biscuits to patients (64 (92.8%)), although two centres restricted biscuits for only treating episodes of hypoglycaemia, or for selected patients with poor nutritional intake. However, biscuits were the only nutrition offered to patients dialysing in 15 (21.7%) of the main dialysis units and in the satellite units of 26 (41.3%) of the main units. All centres offered a patient a drink of water, tea, coffee or juice.

Hot food was more likely to be offered to patients dialysing in Northern Ireland and Scotland compared to England (X^2^ 23.6, *p* < 0.001; and X^2^ 6.8, *p* = 0.009, respectively). Patients dialysing in Wales were not routinely offered hot food. Excluding those centres providing hot food, then 38% of main dialysis units, and 58% of dialysis units with satellite centres in England, did not routinely offer patients a sandwich, whereas all main dialysis units in Wales provided sandwiches, and only one centre did not routinely offer sandwiches for all their satellite units. Similarly, only one centre in both Scotland and Northern Ireland did not regularly provide sandwiches to dialysis patients.

## Comparison to Europe and world-wide

Practices in other European countries differ from the UK, with more countries providing meals, although the majority supplying sandwiches to patients dialysing in main centres (Table 1). Whereas food is not generally provided In North America, most centres in South America, the Middle East and North Africa provide some food (Table 2). Apart from India, most Asian countries do not regularly provide food in government dialysis centres. Meals may be provided in private dialysis centres in Australia and Japan. Whereas some countries which provide no food, such as Austria, Thailand and Singapore encourage patients to bring along their own food, other countries, such as Mexico and many dialysis centres in North America, actively discourage patients from eating during dialysis.

**Table 1:** Food offerings to patients with chronic kidney disease attending for haemodialysis in European countries

Country	Main centre	Satellite dialysis	Comments
Austria	No food		Bring own food
Belgium	Warm food or sandwiches, protein rich puddings	Warm or cold food or sandwiches	
Czech Republic	Sandwiches		
France	Sandwiches	Typically, no food	
Germany	Hot or cold food		
Greece	Sandwiches		
Italy	Sandwiches		
North Macedonia	No food	Sandwiches in private centres	Bring own food
Netherlands	Minority hot food majority snacks	Snack	
Poland	Sandwiches		
Portugal	Sandwiches		
Russian Federation	No food		Private centres biscuits
Spain	Sandwiches		
Sweden	Sandwiches

**Table 2:** Food offerings to patients with chronic kidney disease attending for haemodialysis in the Americas, Africa, Middle East and Asia–Pacific regions. National Kidney Foundation (NKF)

Country	Main centre	Satellite dialysis	Comments
USA	No food	No food	Eating discouraged
Canada	No food	No food	Eating discouraged
Mexico	No food		Eating discouraged
Brazil	Sandwiches or snacks	Snacks	
Chile	Biscuits		Sandwiches preCovid
Columbia	Biscuits post-dialysis	No food in one major chain	Eating discouraged
Egypt	Snacks		
Morocco	No food		Hot meals private centres
Zambia	Porridge/hot meal		Biscuits in private units
Lebanon	Hot or cold meals		
United Arab Emirates	Sandwiches		
Kingdom Saudi Arabia	Meals or sandwiches		
India	Breakfast, hot meal or sandwiches		
Thailand	No food		Bring own food
South Korea	No food		Small centres may offer food
Singapore	No food	NKF biscuits	Bring own food
China	No food		Small and private centres may offer food
Japan	No food		Private centres may offer food, and Food boxes for purchase
Australia	Sandwiches or snacks	Sandwiches or snacks	Private centres may offer meals
New Zealand	No food		Bring own food

### Effect of Covid-19

During the Covid-19 pandemic most dialysis centres stopped providing hot food, and requested patients not to eat during dialysis. Many centres also stopped providing sandwiches, and those which continued to supply sandwiches or snacks gave these to patients on leaving the dialysis units to eat later at home. Most centres have now returned to their pre-pandemic practices, although several of the UK satellite centres have changed to only offering patients biscuits.

## Discussion

The increasing number of elderly frail patients with CKD treated by haemodialysis continues to increase [1], and these patients are at increased risk of sarcopenia and protein energy wasting [3, 5]. Although many clinical guideline committees have recommended that dialysis patients should eat 1–1.2 g/kg/day and 25–35 kcal/kg/day, many elderly patients do not achieve these targets [6], due to a variety of reasons, including psychological and financial factors, as well as medical issues [12]. In addition, studies reviewing dietary records demonstrate that most HD patients eat less protein and calories on their dialysis day compared to the non-dialysis day [13, 14]. Thus, the ISRNM recommended that HD patients should be offered food when they attend for dialysis. [10].

Our survey demonstrated that most centres in the UK offered biscuits, followed by a sandwich, with only a small minority providing a meal. Very few dialysis units provided hot food, apart from two centres which provided hot food to patients in satellite units, this was otherwise only available in the main centres, and in most units restricted to patients identified by dietitians as being at risk of malnutrition. Although the UK has a National Health Service (NHS), there are differences between health care provision in England, Wales, Scotland, and Northern Ireland. Patients were much more likely to be offered hot food when attending dialysis centres in Northern Ireland and Scotland.

There have been concerns that eating a hot meal during a dialysis session potentially increases the risk of intra-dialytic hypotension [15]. As such many centres in Mexico and North America do not offer food during dialysis sessions, and other centres restrict food to critically ill patients, or those with unstable cardiovascular conditions. In theory, patients could also potentially be at risk of aspiration, increased gastrointestinal symptoms, possible hygiene issues especially with the Covid-19 epidemic, along with additional staff workload and increased financial costs. However, there appears to be limited reports of such complications in real-world scenarios [10].

Almost 60% of dialysis units in the UK only offered biscuits to patients dialysing in their satellite dialysis units. Although the type of biscuits varied between units, an average estimate of a mini-pack was around 2.1 g of protein and 200 kcal. After biscuits, a majority offered sandwiches, although some dialysis units restricted this practice to the main centre, and selected patients. Just over a third of English dialysis units did not routinely offer sandwiches to patients dialysing in their satellite units, compared to one or no centres in Wales, Scotland and Northern Ireland.

Compared to other European countries, UK patients were less likely to be offered a warm meal, and as the UK has relatively more satellite dialysis patients, then fewer UK patients would be offered a sandwich. However, world-wide the policy in many countries is not offer patients food when attending for dialysis, and relying on patients to either eat before or after the dialysis session, or bring their own food.

Most UK dialysis centres offered a choice of cheese, ham, chicken, tuna, and egg sandwiches, with an estimated median protein content of 15.5 g (range of 14–18 g), and calorific content of 340 kcal (range 260–413) kcal. A small study from Brazil reported that protein intake dropped from 1.0 g/kg/day to 0.8 g/kg/day, and calorific intake from 21 to 18 kcal/kg/day on the dialysis day [13]. The multi-centre North American HEMO study similarly reported that patients had both lower protein intake on dialysis days (60 vs 65 g/day) and calorific intake (1488 vs 1566 kcal/day) [14]. Taking into consideration, the time spent travelling to and from dialysis units, dialysis session time, most of the average patient’s day is taken up with dialysis. As most patients take time to recover from their dialysis treatment [16], they may not eat on return home, or rely on food prepared by partners or relatives. However, with the increasing number of elderly and frail patients, more are living alone, and even those in nursing or care homes, may arrive home after meal-times have ended. Compared to England, Wales, Scotland and Northern Ireland are less densely populated, and as such dialysis centres may be located further away, with longer travelling times. This may account for the greater provision of hot food in Northern Ireland and Scotland, and greater availability of food at satellite dialysis centres in Wales, Scotland and Northern Ireland compared to England.

Providing food for patients attending for dialysis incurs healthcare costs. Although these may be relatively marginal for dialysis centres embedded within a main hospital, costs will be relatively greater for supplying food to smaller satellite centres including additional costs from transport. Rather than provide food to all patients, several UK centres only offered meals or sandwiches to patients identified by dietitians as suffering from malnutrition. Unless a satellite unit was sited within a hospital setting, most UK satellite centres now only provide biscuits, on the assumption that patients dialysing in satellite centres are healthier and less frail than those dialysing in main hospital centres.

Thus, this UK wide survey would suggest that the majority of dialysis patients are offered less than 25% of the recommended daily protein and calorific intake when attending for treatment, particularly those dialysing in satellite dialysis units. As the dialysis day can be extended by journey times, waiting to start dialysis, and time to recover [17], then providing nutrition when patients come for dialysis is important to enable them to meet nutritional targets, as many patients feel tired post-dialysis [18], and may not eat three meals on the dialysis day [12]. The demographics of the dialysis population in the UK has changed over time to include an increasing number of elderly, co-morbid patients who are at increased risk of sarcopenia and protein energy wasting [3, 5]. As such it is important that dialysis patients are regularly reviewed by renally trained dietitians, as poor nutrition is a key risk factor for patients developing sarcopenia [19], and so reducing nutritional offerings to dialysis patients should be avoided, and centres should take the opportunity to ensure adequate nutritional support to patients when they attend for dialysis.

### Take home message

1. the International Society of Nutrition and Metabolism (ISRNM) recommended that meals or supplements should be considered as a part of the standard-of-care practice for patients attending for haemodialysis
2. Dialysis patients are at increased risk of frailty, sarcopenia and protein energy wasting, and inadequate nutrition risks increasing frailty, sarcopenia and protein energy wasting,
3. Patients may spend many hours travelling to and from dialysis, and time sent in the dialysis centre, and as patients may take time to recover from dialysis, then providing nutrition to patients when they come to the dialysis centre is an important part of their clinical management

## Declarations

### Availability of data and material

UCL department of renal medicine R drive.
